# Effect of Poly-γ-Glutamic Acid Molecular Weight on the Properties of Whey Protein Isolate Hydrogels

**DOI:** 10.3390/polym17121605

**Published:** 2025-06-09

**Authors:** Daniel K. Baines, Zuzanna Pawlak-Likus, Nikoleta N. Tavernaraki, Varvara Platania, Mattia Parati, Timothy N. Wong Wong Cheung, Iza Radecka, Patrycja Domalik-Pyzik, Maria Chatzinikolaidou, Timothy E. L. Douglas

**Affiliations:** 1 School of Engineering, Lancaster University, Gillow Avenue, Lancaster LA1 4YW, UK; d.baines3@lancaster.ac.uk (D.K.B.); t.wongwongcheung@lancaster.ac.uk (T.N.W.W.C.); 2 Department of Biomedical and Life Science, Lancaster University, Gillow Avenue, Lancaster LA1 4YW, UK; 3 Department of Biocybernetics and Biomedical Engineering, Faculty of Electrical Engineering, Automatics, Computer Science and Biomedical Engineering, AGH University of Krakow, 30-059 Krakow, Poland; zpawlak@agh.edu.pl; 4 Department of Materials Science and Engineering, University of Crete, 700 13 Heraklion, Greece; ntav@materials.uoc.gr (N.N.T.); plataniavarvara@yahoo.com (V.P.); mchatzin@materials.uoc.gr (M.C.); 5 Faculty of Science and Engineering, School of Pharmacy and Life Sciences, University of Wolverhampton, Wolverhampton WV1 1LY, UK; m.parati@wlv.ac.uk (M.P.); i.radecka@wlv.ac.uk (I.R.); 6 Department of Biomaterials and Composites, Faculty of Materials Science and Ceramics, AGH University of Krakow, 30-059 Krakow, Poland; pdomalik@agh.edu.pl; 7 Institute of Electronic Structure and Laser, Foundation for Research and Technology Hellas, 700 13 Heraklion, Greece

**Keywords:** whey protein isolate, γ-PGA, polymers, biomaterials, hydrophilic, bone scaffolds, osteogenesis, osteogenic differentiation, tissue engineering

## Abstract

Whey protein isolate (WPI) hydrogel is a promising candidate as a biomaterial for tissue engineering. Previously, WPI hydrogels containing poly-γ-glutamic acid (γ-PGA) with a molecular weight (MW) of 440 kDa demonstrated potential as scaffolds for bone tissue engineering. Here, the study compares different γ-PGA preparations of differing MW. WPI-γ-PGA hydrogels containing 40% WPI and 0%, 2.5%, 5%, 7.5%, and 10% γ-PGA were synthesised. Three γ-PGA MWs were compared, namely 10 kDa, 700 kDa, and 1100 kDa. Evidence of successful γ-PGA incorporation was demonstrated by scanning electron microscopy and Fourier transform infrared spectroscopy. Increasing γ-PGA concentration significantly improved the swelling potential of the hydrogels, as demonstrated by ratio mass increases of between 85 and 90% for each 10% variable group. Results suggested that γ-PGA delayed enzymatic proteolysis, potentially decreasing the rate of degradation. The addition of γ-PGA significantly decreased the Young’s modulus and compressive strength of hydrogels. Dental pulp mesenchymal stem cells proliferated on all hydrogels. The highest cellular growth was observed for the WPI-700 kDa γ-PGA group. Additionally, superior cell attachment was observed on all WPI hydrogels containing γ-PGA compared to the WPI control. These results further suggest the potential of WPI hydrogels containing γ-PGA as biomaterials for bone tissue engineering.

## 1. Introduction

Chronic bone-related pathologies occur when a break or fracture fails to heal correctly, resulting in non-union [1]. The initial fracture can result from trauma or underlying health conditions such as diabetes and osteoporosis [2]. Although bone has the potential to self-repair, approximately 5–10% of fractures fail to heal correctly, resulting in non-union of the bone, requiring sustained treatment [3,4]. Globally, in 2019, there were 455 million cases of acute or non-healing fractures [5]. In 2017, the cost to Europe for fractures was GBP 30.5 billion, a number reportedly expected to rise by 27% in 2030 [6], due mainly to an ageing population [7].

Currently, the gold standard treatment for non-union is an autologous bone graft [8]. However, autologous bone grafts present limitations [9]. For instance, bone harvesting requires an additional surgery [10]. Additionally, there is a limited amount of bone material available [11]. Therefore, there is a necessity for artificial biomaterials as replacements for traditional autologous bone grafts. Numerous approaches have been employed to synthesise scaffold biomaterials for bone regeneration from metals, ceramics, proteins, polysaccharides, or composites [12,13,14,15,16]. However, current scaffolds still present some limitations caused mainly by the nature of the biomaterial used [17]. Ideally, scaffolds should be non-cytotoxic, biodegradable, and porous, offering an increased surface area for cellular attachment and proliferation and promoting cellular proliferation [18]. Additionally, ideal scaffolds would have adequate mechanical properties, including mechanical strength similar to bone and an ability to distribute weight in a similar manner to that of bone [19].

Recently, protein-based hydrogel biomaterials based on whey protein isolate (WPI) have demonstrated numerous desirable properties. WPI is purified from Whey [20], the main waste product from the dairy industry, accounting for approximately 90% of the waste from cheese production [21]. The main protein in WPI is β-lactoglobulin (BLG) [22]. WPI is a suitable scaffold biomaterial as it forms sterilisable hydrogels. Hydrogel formation is induced mainly through heating, which initiates the denaturing of the β-lactoglobulin protein. The denaturing of the proteins allows for the interactions of methionine and cystine residues forming disulphide bridges; hydrophobic–hydrophobic interactions also promote crosslinking between protein molecules [23].

Another advantageous property of WPI is the potential to incorporate biologically active molecules. Previously, WPI hydrogels have been loaded with flower extracts, antibiotics, and small biologically active molecules [24,25]. WPI hydrogels have been enriched with bioactive glass and antimicrobial molecules and have functioned as a drug delivery system for hydrophobic medication [26,27,28]. Additionally, WPI hydrogels have supported cellular attachment, proliferation, and differentiation in multiple investigations [29,30]. Furthermore, WPI hydrogels loaded with a 440 kDa variant of γ-PGA previously supported the growth and osteogenic differentiation potential of MG-3T3 E1 pre-osteoblasts [31].

γ-PGA is a hydrophilic protein-like polymeric substance consisting of a polymer chain of repeating l-glutamic acid and d-glutamic acid monomers [32]. The molecule was first identified in a capsule of *Bacillus antheracis*, consisting of D-γ-PGA homopolymer. Since the initial isolation, γ-PGA has been isolated from other microbes—mainly in the D/L γ-PGA racemic mixture—including *Bacillus licheniformis*, *Bacillus subtilis* subsp. natto, *Rhodopirellula baltica*, and *Staphylococcus epidermidis* [33]. It has been demonstrated that γ-PGA is produced during the citric acid cycle of the microbe [34]. Recently, γ-PGA has demonstrated the potential to be antimicrobial, immunogenic, cytocompatible, conducive to enamel protection, and biodegradable [35,36,37].

**Figure 1:**
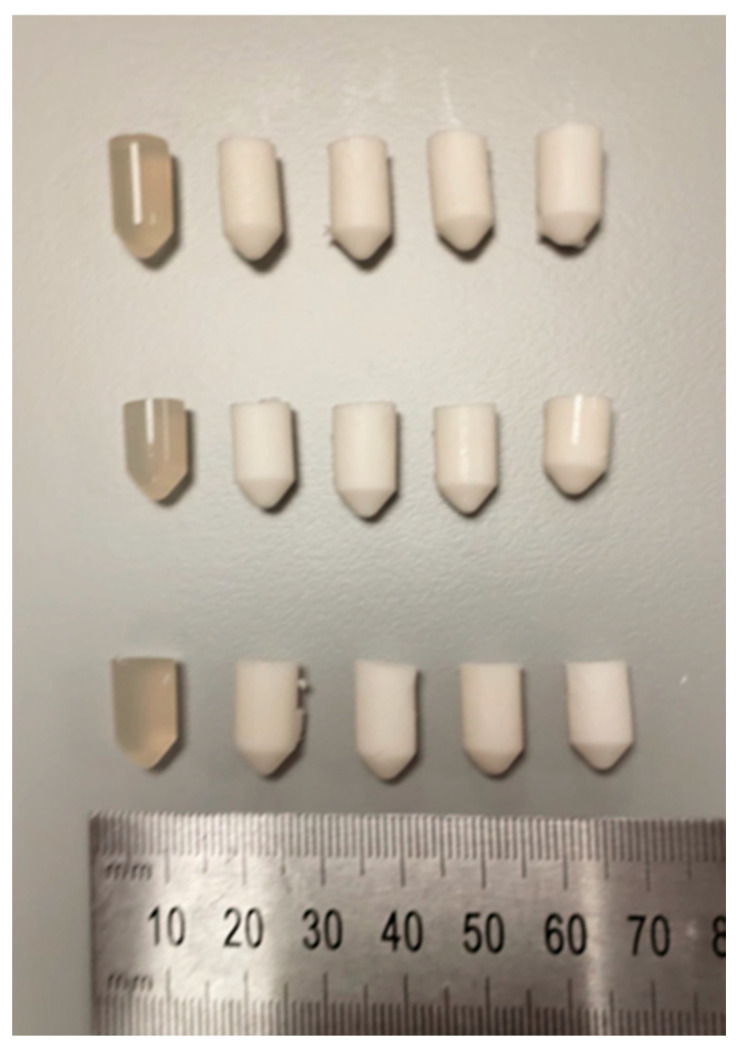
An image of the WPI-γ-PGA hydrogels post-sterilisation. The bottom row is the 10 kDa sample groups. The middle row is the 700 kDa sample groups, and the top row represents the 1100 kDa sample groups.

The investigation sought to combine the advantageous properties of both WPI and γ-PGA by generating and characterising WPI-γ-PGA hydrogels to be utilised as scaffolds for tissue engineering. The aim was to begin the fine-tuning process of the WPI-γ-PGA hydrogels, analysing both degradation and mechanical profiles for tissue regeneration. Three γ-PGA preparations of different MW were compared, namely 10 kDa, 700 kDa, and 1100 kDa. The γ-PGA concentrations were also varied; 2.5%, 5%, 7.5%, and 10% concentrations were compared, Figure 1. To determine degradation profiles, the physiochemical characterisation included investigations of swelling and degradation by proteolytic enzymes at pH 7 and release investigations. Mechanical properties were also analysed. Additionally, biological characterisation, including the determination of cell viability, was performed using dental pulp mesenchymal stem cells (DPSCs).

## 2. Materials and Methods

### 2.1. WPI/Poly-γ-Glutamic Acid Hydrogel Formation

WPI-γ-glutamic acid hydrogels were synthesised according to the following method. WPI solutions were formed at 40% *w*/*v* with deionised H_2_O. The WPI was sourced from (Davis and Co). γ-PGA with a molecular weight of 10 kDa, 700 kDa, and 1100 kDa was added at concentrations of 2.5%, 5%, 7.5%, or 10% to form the WPI-γPGA sample groups, Table 1. The solutions were homogenised for 24 h on an IKA Loopster (IKA England LTD, Oxford, UK). Post-homogenisation, the samples were degassed and formed into 1 mL/1 g samples for analysis. Sterilisation was achieved via autoclaving.

### 2.2. Scanning Elctron Microscopy

Scanning electron microscopy (SEM) was used to determine the successful incorporation of γ-PGA into the hydrogels. The assay was conducted with a JSM-6390 LV, JEOL Ltd., (Welwyn Gardens, UK). scanning electron microscope with an accelerating voltage of 15 kV. The analysis was conducted on four hydrogel sample groups, WPI0 hydrogel control, WPI γ-PGA 10, 10, WPI-γ-PGA 700, 10, and WPI-γ-PGA 10, 10 sample groups, comparing the WPI0 control to the three-maximum concentration WPI-γ-PGA molecular weight sample groups. Dehydrated samples from the centre of the hydrogel were gold coated and imaged and ×100, ×3000, and ×10,000 magnification.

### 2.3. Fourier Transform Spectroscopy

FTIR provided the basis to ascertain the successful synthesis of WPI-γ-PGA hydrogels. The investigation utilised hydrogel samples cut to a thickness of 0.5 mm. The samples were dehydrated, and the spectra were analysed with a Cary 630 FTIR spectrophotometer (Agilent, Santa Clara, CA, USA). A spectral range of between 650 and 4000 cm^−1^ was analysed, with 32 scans per sample.

### 2.4. Polymer Swelling Analysis

Polymer swelling analysis was used to determine the effect of the incorporation of γ-PGA on the structural integrity of the WPI hydrogels. Additionally, the assay sought to investigate the effect of the neutral pH of the osteo environment on the swelling potential of the hydrogels. The method was as follows: WPI-γ-PGA hydrogel samples with a mass of 1 g were placed in 5 mL phosphate-buffered saline (PBS). The samples were incubated at 37 °C for 5 days. The ratio mass change was calculated as a percentage where the swelling percentage (S%) was calculated from the wet mass (Mw) and the dry mass (Md).

S% = (Mw − Md)/Md × 100

### 2.5. Enzymatic Degradation

An enzymatic degradation assay was used to determine the effect of enzymes on the WPI hydrogels. The investigation utilised protease (Sigma, ThermoFisher, Loughborough, UK) to known concentrations of collagenase and was adapted from [38]. The experiment was conducted in a neutral pH environment. The method was as follows: WPI-γ-PGA hydrogel samples with a mass of 1 g were placed in a 5 mL PBS/collagenase solution. The samples were incubated at 37 °C for 5 days. The percentage ratio mass change was calculated where the swelling percentage (S%) was calculated from the wet mass (Mw) and the dry mass (Md).

S% = (Mw − Md)/Md × 100

### 2.6. Release Profiling

UV–Vis spectroscopy was utilised to determine the release of protein over a 5-day period. WPI-γ-PGA hydrogel samples and the WPI0 control samples with a mass of 1 g were treated with 5 mL PBS and incubated for a 5-day period at 37 °C (n = 15). After 5 days, the solute was analysed by UV–Vis spectroscopy using Nanodrop^TM^ (ThermoFisher, Loughborough, UK) with a focus on two wavelengths, namely λ280 nm and λ216 nm.

### 2.7. Mechanical Analysis

Compression analysis was utilised to determine the effect of the addition of the different molecular weight γ-PGA preparations on the structural integrity of the hydrogels. WPI-γ-PGA hydrogels were formed to concentrations consistent with the investigation, with a height of 10 mm and a radius of 4 mm. The compression analysis was conducted with an Instron 3345, Instron, (High Wycombe, UK). The rate of compression was 2 mm/min. The Young’s modulus, compressive strength, and strain at the break of the hydrogels were calculated using the following formulae.

Youngs modulus (Ε) was calculated as follows:

Ε = σ/∈

where σ denotes stress and ε denotes strain.

Compressive strength was calculated as follows:

F = P/(πr^2^)

where P is the load at failure and A is the cross-sectional area.

Strain at break was calculated as follows:

∈ = ∆L/L × 100

where ΔL is the difference between the initial length and the final length and L is the initial length.

### 2.8. Cellular Analysis

#### 2.8.1. Cell Culture and Viability Assay

In vitro cytotoxicity, cell adhesion, and morphology were conducted utilising DPSCs as a model cell type. The cells were cultured in alpha-MEM (PAN-Biotech, Aidenbach, Germany), supplemented with 15% foetal bovine serum (FBS) (PAN-Biotech, Aidenbach, Germany), 2 mM L-glutamine (PAN-Biotech, Aidenbach, Germany), 100 μg/mL penicillin/streptomycin (PAN-Biotech, Aidenbach, Germany), and 2.5 μg/mL amphotericin (Thermo Fisher Scientific, Waltham, MA, USA). The cells were incubated in 5% CO_2_ at 37 °C.

Cells were trypsinised with trypsin/EDTA (Thermo Fisher Scientific, Waltham, MA, USA) and subsequently seeded onto the WPI-γ-PGA scaffolds. Prior to cell seeding, the scaffolds underwent a 10 min UV irradiation. For the proliferation assessment, a suspension of DPSCs (50 × 10^3^ cells per scaffold) was introduced into a 10 μL volume of complete medium. Subsequently, 200 μL of culture medium was added to each scaffold. The culture medium was replaced every three days.

An AlamarBlue™ (Thermo Fisher Scientific, Waltham, MA, USA) viability assay was conducted to evaluate the cellular viability of the WPI-γ-PGA hydrogels. The hydrogels were seeded with DPSCs (n = 5). The resazurin-based indicator stains viable cells, producing a red product that can be analysed photometrically. On days 3 and 5, 200 μL of Al-amarBlue™ (ThermoFisher Scientific, Waltham, MA, USA), reagent, diluted in alpha-MEM at a 1:10 ratio, were added to each well and incubated at 37 °C for 60 min. After incubation, 100 μL of the supernatants were transferred to a 96-well plate, and their absorbance was measured at 570 and 600 nm using a Synergy HTX Multi-Mode Microplate Reader (BioTek, Bad Friedrichshall, Germany). The cell-seeded scaffolds were rinsed twice with PBS, and their culture media were renewed.

#### 2.8.2. Cell Adhesion and Morphology Evaluation with SEM

SEM was used to examine cell attachment and morphology on the WPI-γ-PGA and hydrogels. The evaluation was conducted with a JEOL JSM-6390 LV scanning electron microscope with an accelerating voltage of 15 kV. DPSCs (50 × 10^3^ cells per sample) were seeded onto the hydrogels and incubated at 37 °C in a CO_2_ incubator for 5 days. The hydrogels were then rinsed with PBS and fixed using a 4% *v*/*v* paraformaldehyde solution for 15 min. Following fixation, the hydrogels were dehydrated using a graded ethanol series (30% to 100% *v*/*v*) and subsequently dried with hexamethyldisilane (HMDS) (Sigma-Aldrich, St. Louis, MO, USA) to ensure complete dehydration while preserving their structural integrity. Finally, the samples were coated with a 20 nm thick layer of gold using a sputter coater (Baltec SCD 050, Los Angeles, CA, USA).

### 2.9. Statistical Analysis

Statistical analysis was performed using two-way ANOVA in GraphPad Prism version 8 software to assess the significance of differences among various scaffold compositions and the control at different experimental time periods. A *p*-value (*) less than 0.05 was considered significant, ** *p* < 0.01, *** *p* < 0.001, and **** *p* < 0.0001, compared to the WPI control scaffold at the corresponding time point.

## 3. Results and Discussion

### 3.1. Scanning Electron Microscopy

**Figure 2:**
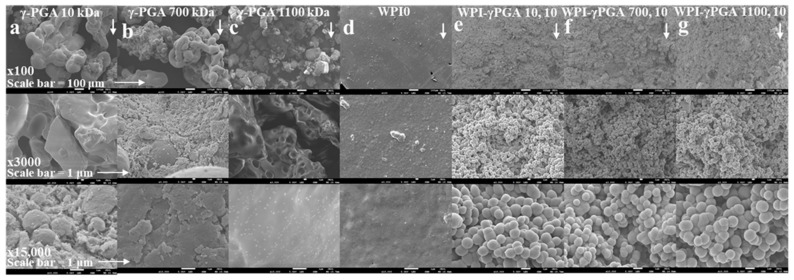
SEM images from left to right of (**a**), γ-PGA 10 kDa, (**b**), γ-PGA 700 kDa, (**c**), γ-PGA 1100 kDa, (**d**), the WPI0 hydrogel control, (**e**), the WPI-γ-PGA 10, 10 sample group, (**f**), the WPI-γ-PGA 700, 10 sample group, and (**g**), the WPI-γ-PGA 1100, 10 sample group. The images were taken at ×100, ×3000, and ×15,000 magnification, the arrows indicate the direction of increased magnification.

Scanning electron microscopy (SEM) was utilised to demonstrate the successful incorporation of γ-PGA into the WPI hydrogel. The resulting images can be observed in Figure 2. The images were obtained at ×100, ×3000, and ×10,000 magnification using the WPI0 control and the 10% γ-PGA sample groups. The WPI0C samples presented mainly a flat, featureless surface at all magnifications. In contrast, the surfaces of samples containing γ-PGA were seen to be textured at ×100 magnification. Further inspections at ×3000 and ×10,000 demonstrated the presence of congruent spheroid clusters.

The analysed samples were taken from a portion of the centre of the hydrogel. Based on the images and the formation of a cluster of spherical shapes, the results suggest changes in the hydrogel formation and potential interactions between WPI and γ-PGA, especially when compared to the γ-PGA kDa controls. The MW of the γ-PGA appeared to have no marked effect on the size of the spheres, which were approximately 1 μm in diameter. There are potential explanations for the formation of the clusters. For example, the charges of both WPI and γ-PGA can lead to electrostatic complexation and phase separation, resulting in coacervation or complex coacervation, which in turn can result in spheres and clusters of spheres. Similarly, the spheres could be formed by the thermal aggregation of the WPI, where the denatured WPI is stabilized and formed into spheres through interactions with the γ-PGA chains, perhaps due to exposure of the hydrophobic regions or the exposure of the reactive groups. However, discussion of the exact nature of this interaction must remain speculative in view of the limited data available.

### 3.2. Fourier Transform Infrared (FTIR) Spectroscopy

**Figure 3:**
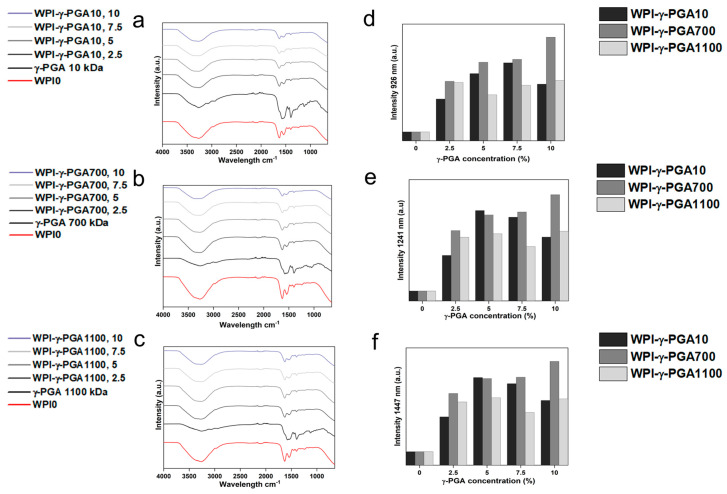
FTIR results of the various WPI-γ-PGA sample groups (a.u.): the 3 MW γ-PGA powders, (**a**), the WPI-γ-PGA 10 kDa, (**b**), WPI-γ-PGA 700 kDa, and (**c**), WPI-γ-PGA 1100 kDa (intensity in arbitrary units (a.u.) with wavenumber), while (**d**–**f**) represent the intensity at wavelengths 926, 1241, and 1459 cm^−1^, respectively. Each wavelength represents the mean of n = 3.

FTIR analysis results can be seen in Figure 3a–c. Post-baseline removal, the hydrogels demonstrated main bands at 3282 cm^−1^, 1630 cm^−1^, 1546 cm^−1^, 1459 cm^−1^, 1397 cm^−1^, and 1241 cm^−1^. The amide I, II, and III regions were observable at the wavenumbers 1630 nm, 1546 nm, and 1241 nm, respectively. An overview of underlying interactions is presented in Table 2.

To study possible interactions between γ-PGA and WPI, further analysis focused on regions of interest known to be associated with glutamic acid. The underlying glutamic acid interactions can be observed in Table 3. For instance, [39] suggested glutamic acid side chain interactions at 926 cm^−1^, a result of C-C stretching vibrations. The results visible in Figure 3e,f demonstrated significant changes in this region, increased in the samples containing γ-PGA. Similarly, other regions of interest demonstrated similar results. Intensity for all γ-PGA concentrations increased at 1241 cm^−1^ when compared to the WPI0 control. The region 1241 cm^−1^ could be associated with glutamic acid side chain stretching vibrations of C-O bonds, side chain twisting vibrations of CH_2,_ and in-plane bending vibrations of C-H bonds. Likewise, the region at 1447 cm^−1^ showed an increase in intensity for all concentrations when compared to the WPI control.

### 3.3. Hydrogel Swelling Analysis

**Figure 4:**
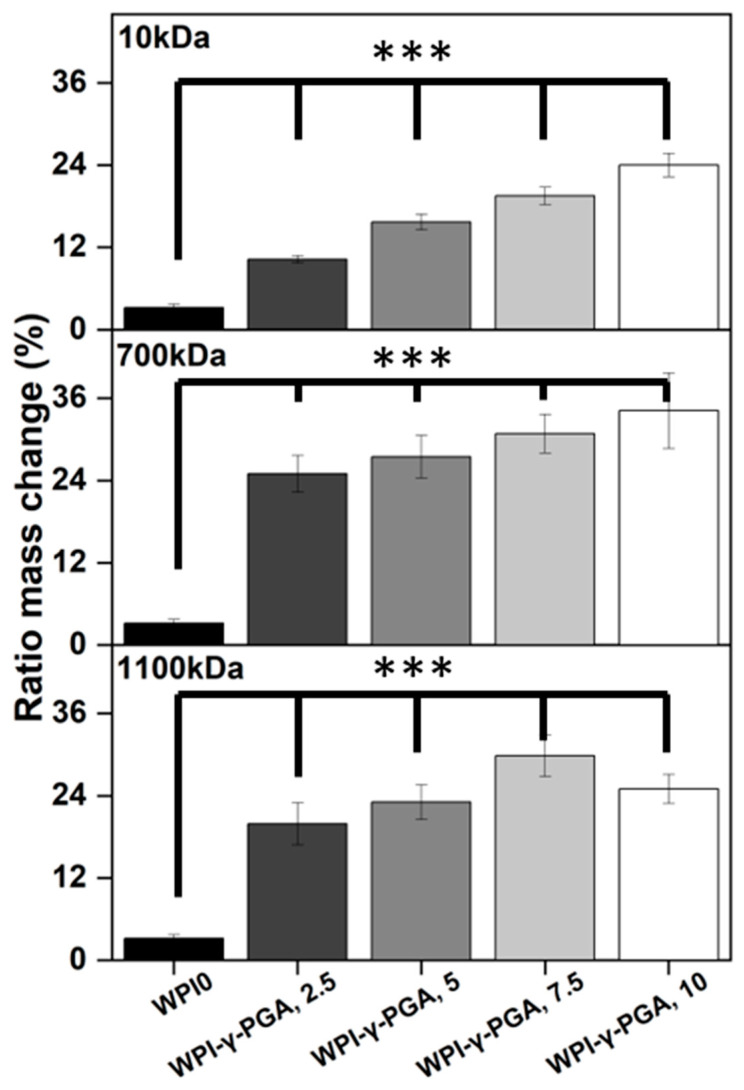
Results of swelling assays. Hydrogel samples were incubated at pH 7 for 5 days, and the ratio mass change as a percentage was calculated. Each bar represents the mean ± SD of n = 10 (*** *p* < 0.001 compared to the WPI control).

Hydrogel swelling analysis was conducted to determine the effect of γ-PGA; the results can be seen in Figure 4. The hydrogels were introduced in a pH environment consistent with a bone environment (pH 7). The introduction of γ-PGA to the WPI hydrogels significantly increased the swelling of the hydrogels for all γ-PGA molecular weights and incremental concentrations (*p* < 0.05). However, there was one exception, namely the WPI1100-γ-PGA, 10% sample group, which swelled less than the WPI-γ-PGA 1100, 7.5%.

The increase in swelling due to the addition of γ-PGA was to be expected, mainly due to the hydrophilic nature of γ-PGA. However, in contrast to our previous investigation [31], in which 440 kDa γ-PGA was utilised, greater swelling was observed for all percentage concentrations for the WPI-γ-PGA 10 kDa, WPI-γ-PGA 700 kDa, and WPI-γ-PGA 1100 kDa sample groups. The maximum swelling for the WPI-10 kDa γ-PGA hydrogels was observed in the 10% sample group; mass increased by a factor of 7.4 when compared to the WPI0 control. Similarly, the maximum ratio mass change for the WPI-700 kDa γ-PGA hydrogels was observed for the 10% sample group, which demonstrated an increase in mass by a factor of 10.7 when compared to the WPI control. Previously, the addition of γ-PGA with a molecular weight of 440 kDa improved the swelling until the concentration of 10% γ-PGA was reached, whereupon the ratio mass change began to decrease. In this investigation, only the WPI-γ-PGA 1100 sample groups followed the same trend. For instance, the largest mass increase for the WPI-γ-PGA 1100 kDa hydrogels was the WPI-γ-PGA 1100, 7.5% sample group, which demonstrated an increase in mass by a factor of 9.3 compared to the increase in mass of a factor of 7.8 achieved by the WPI-γ-PGA 1100, 10% sample group when compared to the WPI control, Figure 4. Both the 10 kDa and 1100 kDa γ-PGA were known to present similar molar mass, potentially the reason behind the similar results, when contrasted with the WPI-γ-PGA 700. However, it should be stated that the relative concentration of the L and D isomers of γ-PGA could potentially have an influence on the results. It would be expected that the addition of γ-PGA beyond a certain concentration would impact the structural integrity of the hydrogels, which would be demonstrated by a decrease in ratio mass change due to the increased degradation of the hydrogels. This could potentially be attributed to the main bonding mechanisms involved in the gelation of WPI hydrogels.

WPI hydrogels are formed by disulphide bridges and hydrophobic interactions between β-lactoglobulin molecules. As previously discussed, γ-PGA is a hydrophilic polymer of glutamic acid. Therefore, an increase in glutamic acid decreases the percentage of amino acids that can form disulphide bonds and form hydrophobic interactions. A decrease in both disulphide bridges and hydrophobic interactions decreases the percentage of crosslinks in the hydrogel, potentially increasing the rate of degradation. Overall, the WPI-γ-PGA 700 kDa hydrogel demonstrated a higher swelling potential than both the WPI-γ-PGA 10 hydrogels and the WPI-γ-PGA 1100 kDa γ-PGA hydrogels. Additionally, as demonstrated by the SEM analysis, there is an interaction between the WPI and γ-PGA that may impact the internal structure of the hydrogels.

### 3.4. Enzymatic Degradation

**Figure 5:**
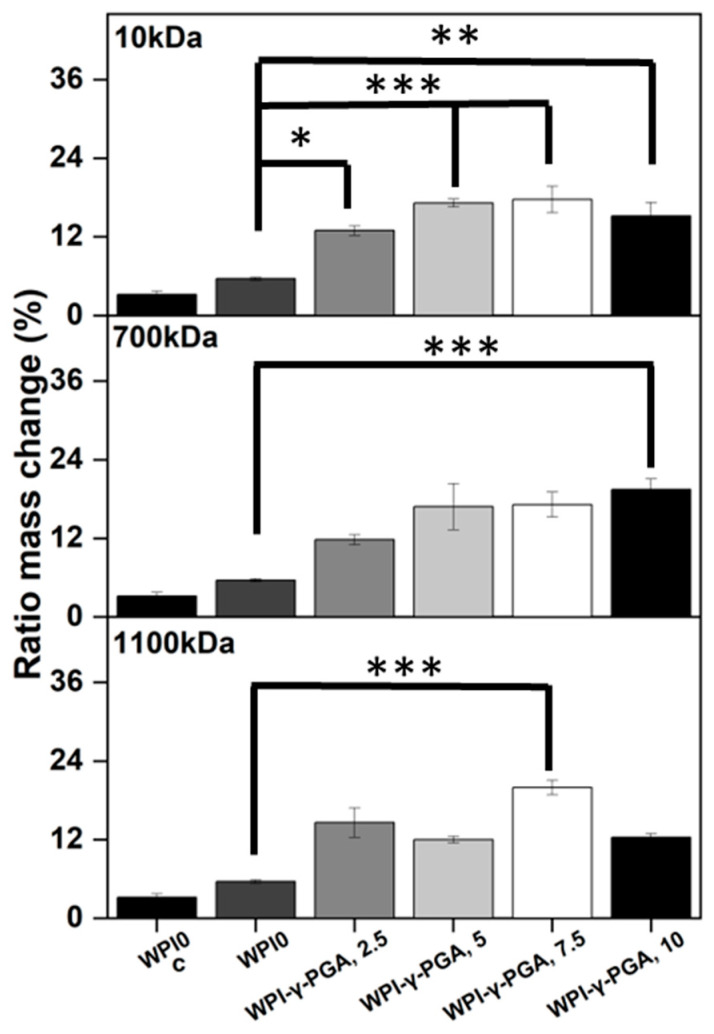
The enzymatic degradation of hydrogel samples incubated at pH 7 with proteases for 5 days and the mass ratio as a percentage. The WPI0c sample group is a control without enzymes, whereas the WPI0 is the 40% WPI hydrogel control with enzymes in the solution. Each bar represents the mean ± SD of n = 10 (* *p* < 0.05, ** *p* < 0.01, *** *p* < 0.001 compared to the WPI control).

The utilisation of protein-based WPI-γ-PGA hydrogels as a tissue engineering biomaterial would expose the hydrogels to potential proteolysis from endogenous proteolytic enzymes [41]. Therefore, an assay was conducted to establish the effect of enzymes on the protein-based hydrogels. The addition of γ-PGA to WPI hydrogels increased the mass ratio change when compared to the WPI controls (*p* < 0.05). The results can be seen in Figure 5.

The increase in mass change was evident for all γ-PGA molecular weight sample groups when compared to the WPI control. However, the trend between each percentage concentration was not always linear, which was potentially the result of imperfections in the hydrogels from the manufacturing process. The WPI-γ-PGA 10kDa hydrogel sample groups displayed a linear increase until the final WPI-γ-PGA10, 10% sample group, as demonstrated by a ratio mass change by a factor of 4.7 when compared to the WPI0 control (*p* < 0.05). The WPI-γ-PGA 700 kDa sample groups demonstrated a linear increase in ratio mass change for all percentage concentrations when compared to the WPI controls. In contrast, the WPI-γ-PGA 1100 kDa sample groups demonstrated fluctuating results, with the WPI-γ-PGA1100 7.5 sample group outperforming the WPI-γ-PGA1100, 10 sample group, presenting an increase in mass by a factor of 6.3 compared to a factor of 2.2 when comparing both to the WPI0 control. The same trend was also observable in the polymer swelling assay for the WPI-γ-PGA 1100 kDa hydrogel sample groups.

When compared to the swelling assay, the hydrogel introduced to enzymes displayed more degradation, the result of the effect of the proteolytic enzymes. However, generally, as the γ-PGA concentration increases, the hydrogels degrade less. The potential explanation could be due to the properties of both WPI hydrogels and glutamic acid. WPI hydrogels are formed mainly through disulphide bonds. However, glutamic acid forms isopeptide bonds by covalent bonds, creating an amide linkage between the side chain or main chain of glutamic acid and the amino group of a lysine side chain. Being outside the main protein chain, isopeptide bonds have a higher chemical stability. Additionally, isopeptide bonds have been demonstrated to be less prone to enzymatic proteolysis than peptide bonds and disulphide bonds formed by the reactive amino acid cystine [42]. Therefore, an increase in γ-PGA decreases the percentage of the more cleavable peptide bonds and disulphide bridges, creating a hydrogel less prone to proteolysis. When the FTIR results are analysed at 1550 cm^−1^, an amide II region associated with isopeptide bonds, there is an observable increase in intensity related to the addition of γ-PGA into the hydrogels, when compared to the WPI control, suggesting that the addition of γ-PGA to WPI increases the number of isopeptide bonds. Furthermore, as γ-PGA has gamma-peptide bonds, it should not be susceptible to alpha-proteases. Previously, isopeptide bonds have been added to antimicrobial peptides to protect from proteolysis [43].

### 3.5. γ-PGA Release

**Figure 6:**
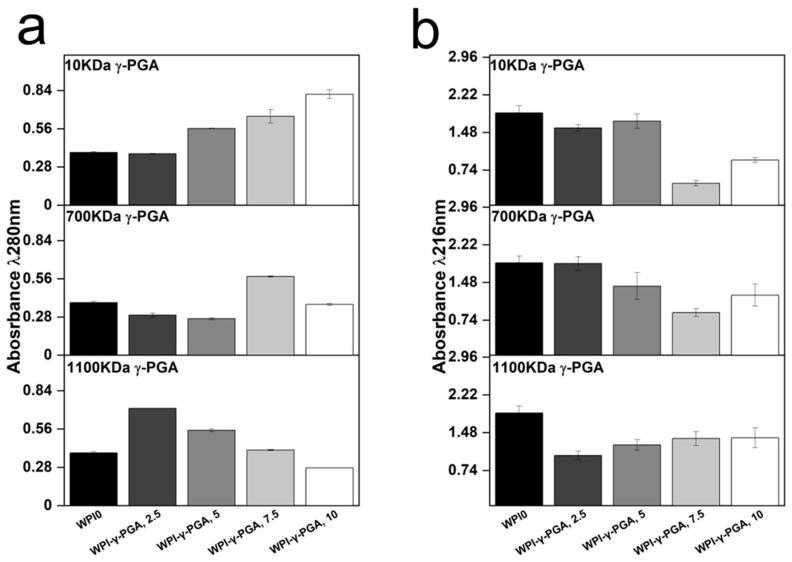
The protein release at (**a**), 280 nm and (**b**), 216 nm. The hydrogel samples were incubated at 37 °C in pH 7 for 5 days, and the solutions were analysed with UV/vis spectroscopy with an emphasis on the 280 nm and 216 nm regions.

Release profiling was utilised to determine the effect on protein release from the hydrogels. The results could provide information on the release of γ-PGA from the hydrogels and the effect of γ-PGA on the stability of hydrogel formation. Two wavelengths were identified as regions of interest, 216 nm, and 280 nm. Analysis at 280 nm is the standard protein wavelength, whereas 216 nm has been associated with γ-PGA [44]. The results can be seen in Figure 6.

The results suggested that the molar mass of γ-PGA exerted opposing effects on the hydrogels when protein release was analysed. For instance, the absorbance at 280 nm increased in a linear fashion with γ-PGA concentration in the 10 kDa γ-PGA hydrogels. Overall, the analysis resulted in a 52% difference in absorbance in the 10% WPI-γ-PGA, WPIPG1010 sample groups when compared to the WPI0 control. However, no significant difference in protein release was observed for the 700 kDa γ-PGA hydrogels when compared to the WPI0 control. In contrast, the 1100 γ-PGA sample groups significantly increased in absorbance for the 2.5% γ-PGA sample group, presenting an absorbance difference of 46% when compared to the control. However, in correlation with an increasing γ-PGA concentration, the absorbance decreased in a linear fashion, resulting in a negative absorbance when compared to the WPI0 control.

The results suggest that the MW of γ-PGA affects the protein release. However, the results could potentially be attributed to an increase in molecule size, overshadowing the WPI protein in the solution. Additionally, the dispersity of the γ-PGA could be having an effect. The 280 nm region is analysed for protein based mainly on interaction from tryptophan and tyrosine residues with some interactions from phenylalanine. The increase in concentration could potentially cause the overshadowing of the Trp and Tyr residues and would be displayed as a decrease in absorbance in the 280 nm region, explaining the result for the 1100 kDa sample groups. Likewise, for the 10 kDa sample groups, the opposite was observed. The decrease in the MW of γ-PGA presents a higher absorbance of the larger MW constituent proteins of WPI. However, the results observed are likely the results of the binding of WPI and γ-PGA and potentially the binding of WPI within the polymer chain of the 1100 kDa γ-PGA.

Analysis of the 216 nm region demonstrated a decrease in absorbance for γ-PGA sample groups when compared to the WPI0 control. This is significant as the 216 nm wavelength was chosen for the identification of γ-PGA. Therefore, should the results at 280 nm be due to overshadowing by the γ-PGA molecule, then the same would be expected in the 216 nm region. However, the opposite is observed, and the WPI0 control shows higher absorbance than the γ-PGA sample groups. Interestingly, the γ-PGA sample group measurements at 216 nm were in contrast to the counterpart results at 280 nm. For instance, the WPI-10 kDa γ-PGA hydrogel sample groups increased in absorbance at 280 nm but decreased at 216 nm. In the WPI-γ-PGA 700 kDa γ-PGA the WPI-γ-PGA 700, 2.5%, WPI-γ-PGA 700, 5%, and the WPI-γ-PGA 700, 10% sample groups, contrasting results were observed at 280 nm and 216 nm. Additionally, the absorbance values for the WPI-1100 kDa γ-PGA sample group decreased with increasing γ-PGA concentration. However, at 216 nm, the absorbance increased, as expected. Therefore, these results suggest that the γ-PGA MW influences the release of WPI native proteins. However, further investigation should be undertaken to determine the effect the enantiomeric ratio of the D and L isomers had on the results. It should be stated that results between 1 and 2 demonstrate less accuracy than those below 1 due to limitations suggested by Beer’s law. Therefore, the results taken at 216 nm could present a higher absorbance reading than expected. However, the results generated some linearity, potentially demonstrating the reliability of the results.

### 3.6. Mechanical Analysis

**Figure 7:**
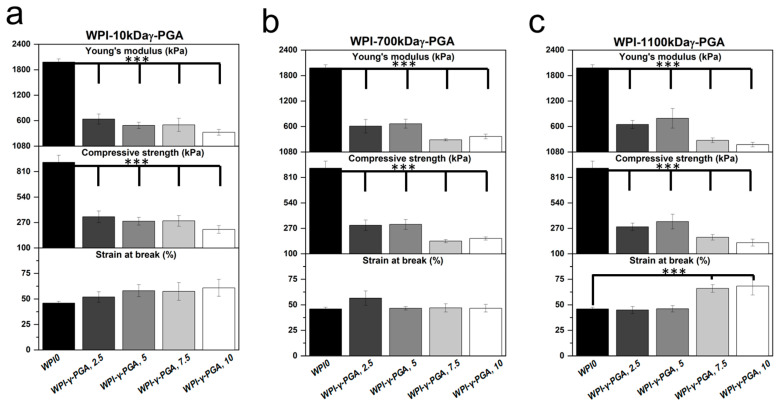
The results of the mechanical testing for the WPI-γ-PGA hydrogels: (**a**), represents the 10 kDa sample groups, (**b**), represents the 700 kDa sample groups, and (**c**), represents the 1100 kDa sample groups. The Young’s modulus, compressive strength, and strain at break were analysed for each MW and concentration sample group. Each bar represents the mean ± SD of n = 5 (*** *p* < 0.001).

Mechanical compressive analysis was used to determine the load-bearing potential of the WPI-γ-PGA hydrogel. The investigation analysed three main parameters: Young’s modulus, compressive strength, and the strain at the break of the hydrogels. The results are displayed in Figure 7. The results demonstrated that the addition of γ-PGA to the WPI hydrogels markedly reduced the mechanical properties of the hydrogels. For instance, the Young’s modulus was reduced by circa 85% for the WPI-γ-PGA 10 kDa sample groups, circa 82% for the WPI-γ-PGA 700 kDa sample groups, and 82% for the WPI-γ-PGA 1100 kDa sample groups (*p* < 0.05). The reduction in mechanical properties due to the addition of γ-PGA could be linked with the hydrogel formation process. WPI hydrogels are formed through interactions between the sulphur-containing amino acids methionine and cystine, forming disulphide bridges. Additional crosslinks are formed through hydrophobic interactions. γ-PGA is a polymer chain of glutamic acid, which makes it highly hydrophilic [43]. Therefore, the addition of γ-PGA to WPI may have impeded the formation of potential disulphide bridges and hydrophobic interactions, weakening the structural integrity of the hydrogels. However, in a practical situation, mechanical movement would be limited. Overall, the WPI-γ-PGA hydrogels formed in this investigation presented inferior mechanical strength when compared to the WPI-γ-PGA hydrogels previously described in [31]. Taking into account the SEM analysis, there is likely an interaction between WPI and γ-PGA, which influences the crosslinking. If there are fewer and/or weaker bonds formed between beta-lactoglobulin molecules, the hydrogels are weaker (Figure 7) and swell more (Figure 4). The discrepancy between the result of the previous 440 kDa variant, which improved the mechanical strength of the WPI-γ-PGA hydrogels, and the results in this study would suggest that MW is potentially not the only defining factor in the functionality of γ-PGA. γ-PGA can be synthesised by an array of microorganisms; its properties are greatly affected by the organism’s cultivation conditions, media composition, and downstream processing. Furthermore, the potential effect, or lack thereof, elicited by γ-PGA can be modified depending on the presence of other compounds, such as WPI, in the case of this manuscript. Additionally, WPI can also demonstrate batch variance. The variance can affect the binding potential of the hydrogels and thus influence such factors as mechanical strength. Therefore, further investigation is required to determine the underlying interactions during the hydrogel formation process and batch variation.

### 3.7. Cellular Analysis

#### 3.7.1. Cell Viability Assay

**Figure 8:**
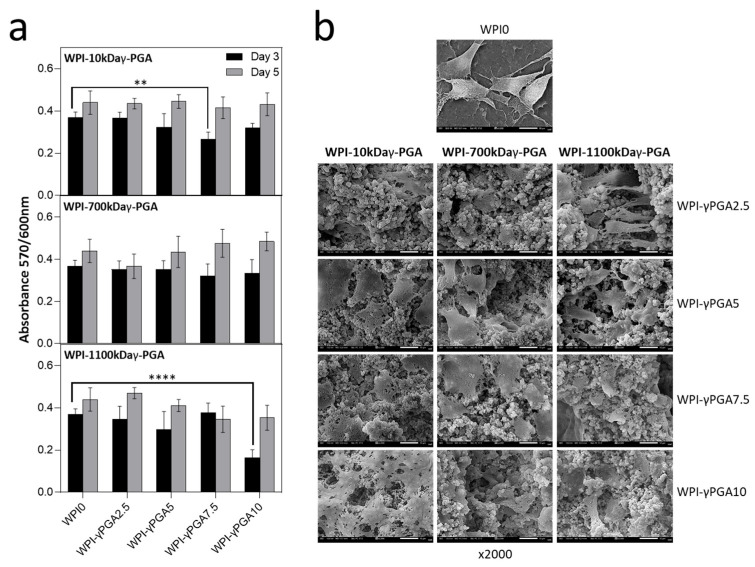
The results of cell viability and proliferation of DPSCs seeded on WPI-γ-PGA hydrogels. (**a**) Absorbance readings at 570/600 nm were taken on days 3 and 5. Each bar represents the mean ± SD of n = 5 (** *p* < 0.01, **** *p* < 0.0001; compared to the WPI0 control). (**b**) SEM images demonstrating the cellular morphology of DPSCs on WPI-γ-PGA hydrogels in 2000× magnification. Images were taken after 5 days in culture. The scale bar represents 10 μm.

In vitro analysis was conducted to evaluate the cytocompatibility of WPI-γ-PGA hydrogel samples using DPSCs. The cytocompatibility was assessed on days 3 and 5 in culture using the AlamarBlue™ assay (Figure 8a). On day 3, a decrease in cell viability was observed across all γ-PGA-containing scaffolds compared to the WPI control. For the γ-PGA 10 kDa variant, the most pronounced decrease in absorbance values was observed at the 7.5% concentration. By day 5, all concentrations exhibited an increase in absorbance values, with no statistically significant differences compared to the WPI control.

A similar trend was noted for the γ-PGA 700 kDa variant, where absorbance values initially decreased as the concentration increased compared to WPI control. However, by day 5, the highest absorbance values were observed for the scaffold with the highest γ-PGA concentration, although no significant differences were noted.

For the γ-PGA 1100 kDa variant, the absorbance values followed a similar pattern, but overall levels were lower than those of the previous two compositions. The most significant difference was observed at the 10% concentration, which had the lowest absorbance values compared to the WPI control. By day 5, most concentrations showed an increase in absorbance, with the exception of the 7.5% concentration. Similar cell viability data at an early examined time period have been previously reported on WPI-γ-PGA hydrogels with a molecular weight of γ-PGA being 440 kDa, as previously discussed in [28].

Notably, reduced cell viability was observed for specific γ-PGA-WPI hydrogel compositions on day 3, particularly in the 10 kDa γ-PGA at 7.5% and 1100 kDa γ-PGA at 10% sample groups. A possible explanation for the lower viability with 10 kDa γ-PGA may be its higher solubility, which can lead to the release of free carboxyl groups and local acidification, transiently affecting cellular metabolism and adhesion. In contrast, the reduced viability observed in the 1100 kDa γ-PGA group at high concentrations may result from an increased matrix density and reduced porosity, which may impair nutrient and oxygen diffusion. These findings highlight a correlation between γ-PGA molecular weight, its concentration, and cell viability, suggesting that scaffold composition should be optimised to maintain biological compatibility while achieving the desired structural and physicochemical properties.

#### 3.7.2. Cell Adhesion and Morphology

The morphology of DPSCs was evaluated by means of SEM imaging at 2000× magnification after 5 days in culture (Figure 8b). DPSCs cultured on WPI-γ-PGA hydrogel compositions exhibited a strong attachment. By day 5, the formation of dense cell layers and cell–cell interactions was observed, which are expected to promote tissue formation. All scaffold groups demonstrated stronger cell attachment compared to the WPI control. In contrast, cells on the WPI hydrogel appeared more spherical in shape and exhibited limited spreading.

## 4. Conclusions

This investigation was undertaken to develop WPI hydrogels with γ-PGA of varying molecular weights. The aim was to begin the process of fine-tuning the WPI-γ-PGA hydrogels to be utilised for bone tissue engineering scaffolds and build upon a previous investigation that utilised a 440 kDa γ-PGA variant. Here, the investigation synthesised WPI-γ-PGA hydrogels with a molecular weight of 10 kDa, 700 kDa, and 1100 kDa, respectively. Both SEM and FTIR analysis suggest the successful incorporation of γ-PGA into the WPI hydrogels for all sample groups. Interestingly, SEM analysis demonstrated the development of spheres, formed by some yet unknown interaction between the WPI and γ-PGA, during the formation process, presenting a route for further investigation. If the spherical aggregates can be consistently induced and a self-assembly mechanism can be established, further drug encapsulation could be possible.

With regards to the investigation of hydrogels in an environment that, to some extent, mimics a biologically relevant one, the addition of γ-PGA led to swelling in a neutral pH environment, providing a surface area increase for cellular attachment. The WPI-γ-PGA hydrogels were shown to be degradable by proteolysis, an advantageous property for a non-toxic hydrogel, with the results potentially beginning to form the basis for timed or predictive degradation. One negative aspect unveiled by this investigation was the decrease in mechanical properties obtained by the hydrogels with the introduction of γ-PGA. The addition of γ-PGA to the WPI hydrogels significantly decreased their Young’s modulus for all γ-PGA molecular weight sample groups. When contrasted with the 440 kDa variant, this would suggest that mechanical strength is impacted by more than the MW of the γ-PGA. However, importantly, biological analysis suggested the proliferation of DPSCs, with the 700 kDa sample groups demonstrating the best results. Therefore, the investigation further suggests the candidacy of WPI-γ-PGA for tissue engineering scaffolding.

## 5. Future Implications and Directions

The future implication of this work results from the potential to develop tissue engineering products fine-tuned to the relevant application, with predictable degradation rates and mechanical properties. These results suggest predictable degradation and demonstrate a potential route to tune mechanical strength. However, it should be stated that the investigation suggested that there are more underpinning factors regarding the binding of WPI and γ-PGA during the formation process. These underlying factors introduce variance—the effect of the variance should be investigated. Additionally, the formation of spheres, as demonstrated in the SEM images, could potentially suggest a route to develop spheres for further drug encapsulation. Further work should elucidate the underlying mechanisms behind the formation of the spheres and a method to produce the spheres constantly. In addition, cell differentiation should be studied in more detail.

## Figures and Tables

**Table 1:** The WPI-γ-PGA hydrogel sample groups and their constituent concentrations.

Sample Name	γ-PGA MW	WPI%	γ-PGA Concentration (%)
WPI0	0	40	0
WPI-γ-PGA 10, 2.5	10	40	2.5
WPI-γ-PGA 10, 5	10	40	5
WPI-γ-PGA 10, 7.5	10	40	7.5
WPI-γ-PGA 10, 10	10	40	10
WPI-γ-PGA 700, 2.5	700	40	2.5
WPI-γ-PGA 700, 5	700	40	5
WPI-γ-PGA 700, 7.5	700	40	7.5
WPI-γ-PGA 700, 10	700	40	10
WPI-γ-PGA 1100, 2.5	1100	40	2.5
WPI-γ-PGA 1100, 5	1100	40	5
WPI-γ-PGA 1100, 7.5	1100	40	7.5
WPI γ-PGA 1100, 10	1100	40	10

**Table 2:** The wavenumbers were acquired by FTIR and the potential underlying interactions. The data were compiled from [39,40].

Wavenumber cm^−1^	Region/Potential Interactions
3281	Amide A—O-H and N-H stretching
1630	Amide I
	Arginine side chain symmetric stretching vibrations—CN_3_H_5+_
	Asparagine side chain in-plane bending vibrations—NH_2_
	Glutamine side chain in-plane bending vibrations—NH_2_
	Lysine side chain antisymmetric in-plane bending vibrations—NH_3+_
	Tryptophan side chain stretching vibration CC, stretching vibration C=C, NH
	Tyrosine side chain stretching vibrations CC ring, in-plane bending vibrations CH
1546	Amide II
	Tyrosine—OH, CC stretching vibrations, CH in-plane bending vibrations
	Lysine side chain interaction—symmetric in-plane bending vibrations—NH_3+_
	Tryptophan—stretching vibration—CN, in-plane bending vibration CH, NH
	stretching vibrations CC ring, in-plane bending vibrations CH
1459	Proline—CN stretching vibrations, CH_2_ in-plane bending vibrations, CH_3_ antisymmetric bending vibrations
	Glutamic acid side chain interactions—in-plane bending vibrations—CH_2_
	Glutamic acid side chain interactions—in-plane bending vibrations—CH_3_
	Glutamine side chain interactions—in-plane bending vibration—CH_2_
	Histidine side chain interactions—in-plane bending vibrations CH, stretching vibrations CN
	Lysine side chain interactions—in-plane bending vibrations—CH_2_
	Proline side chain interactions—in-plane bending vibrations—CH_2_
	Serine side chain interaction—in-plane bending vibrations—CH_2_
	Tryptophan side chain interaction—in-plane bending vibration—NH, stretching vibration—CC, in-plane bending vibration CH
	Tryptophan side chain interactions in-plane bending vibration—CH, stretching vibration—CC, CN
	Tyrosine side chain interactions in-plane bending vibrations—CH_2_
1397	Aspartic acid and glutamic acid—in-plane bending vibrations
	Aspartic acid side chain interaction—symmetric stretching—COO^−^, COH
	Glutamic acid side chain interactions—wagging vibrations—CH_2_
	Threonine—in-plane bending vibrations—COH, CH
	Tyrosine side chain interaction—wagging vibration—CH_2_
1241	Amide III
	Tyrosine side chain interactions—in-plane bending vibrations—COH
	Histidine interactions—in-plane bending vibrations CH, stretching vibrations—CN, and in-plane bending vibrations—NH
	Glutamic acid side chain interactions—stretching vibrations—C-O
	Glutamic acid side chain interactions—twisting vibrations—CH_2_, in-plane bending vibrations—CH
	Histidine side chain interactions—stretching vibrations—CN
	Threonine side chain interactions—in-plane bending vibrations—COH, CH
	Tryptophan side chain interactions—twisting vibrations—CH2, in-plane bending vibrations—CH
	Tyrosine—stretching vibration—CO, stretching vibrations—CC

**Table 3:** The wavenumber associated with glutamic acid. The data were compiled from [31,40].

Wavenumber cm^−1^	Potential Cause of Interaction	Wavelength cm^−1^ in Water
926	Glutamic acid side chain interactions—stretching vibrations—CC	926
1079	Glutamic acid side chain interaction—stretching vibration—CC	COO-1074 COOH 1083
1161	Glutamic acid side chain interactions—stretching vibrations—C-O	COOH 1120-1253
1241	Glutamic acid side chain interactions—stretching vibrations—C-O	COOH 1120-1253
	Glutamic acid side chain interactions—twisting vibrations—CH_2_, in-plane bending vibrations—CH	COO-1225
1397	Glutamic acid side chain interactions—wagging vibrations—CH_2_	COOH 1388
1459	Glutamic acid side chain interactions—in-plane bending vibrations—CH_2_	1452, COOH 1451
	Glutamic acid side chain interactions—in-plane bending vibrations—CH_3_	1440

## Data Availability

Data is contained within the article.
